# Restricted Daily Exposure of Environmental Enrichment: Bridging the Practical Gap from Animal Studies to Human Application

**DOI:** 10.3390/ijerph21121584

**Published:** 2024-11-27

**Authors:** Nik Nasihah Nik Ramli, Nurin Amalia Kamarul Sahrin, Siti Nur Atiqah Zulaikah Nasarudin, Mohamad Hisham Hashim, Maisarah Abdul Mutalib, Muhammad Najib Mohamad Alwi, Aswir Abd Rashed, Rajesh Ramasamy

**Affiliations:** 1 School of Graduate Studies, Management and Science University, Shah Alam 40100, Selangor, Malaysia; 2 International Medical School, Management and Science University, Shah Alam 40100, Selangor, Malaysia; 3 Nutrition, Metabolism and Cardiovascular Research Centre, Institute for Medical Research, National Institutes of Health, Ministry of Health, Malaysia, No.1, Jalan Setia Murni U13/52, Seksyen U13 Setia Alam, Shah Alam 40170, Selangor, Malaysia; 4 Department of Pathology, Faculty of Medicine and Health Sciences, Universiti Putra Malaysia, Serdang 43400, Selangor, Malaysia

**Keywords:** environmental enrichment, restricted, short term, daily, translation

## Abstract

Daily restricted environmental enrichment (REE) refers to limited, structured periods of enrichment aimed at improving both physical and cognitive well-being in animals and humans. This review explores the significance of REE, focusing on studies that investigate 2 and 3 h daily enrichment protocols. Through an analysis of 21 key studies, this paper highlights how even brief periods of REE can lead to substantial improvements in brain plasticity, cognitive function, and stress resilience. The review tracks the evolution of environmental enrichment from early research on enriched environments in animals to modern applications in human rehabilitation, particularly for stroke recovery and mental health treatment. While the traditional approach to environmental enrichment often involves continuous exposure, recent research suggests that restricted daily enrichment can yield comparable benefits, offering a practical, scalable solution for clinical settings. This review underscores the importance of adapting REE for individual needs and developing flexible, home-based programs for broader application.

## 1. Introduction

Environmental enrichment (EE) involves the intentional creation of settings that promote both the physical and psychological well-being of animals. By encouraging the expression of natural behaviors—instinctual, species-specific activities like foraging, exploring, and socializing which animals would naturally exhibit within their habitats—EE aims to support the animals’ overall welfare. Such behaviors are essential not only for maintaining physical health but also for providing cognitive stimulation, as they engage animals in processes that are crucial for their mental enrichment and adaptive capacities within enriched environments [1]. It is also described using terms such as “experience-dependent”, “physical activity”, “cognitive stimulation”, “socialization”, “behavioral diversity”, and “environmental enhancement” [2]. The primary aim of EE is to create environments that encourage desirable species-specific behaviors, contributing positively to the animals’ mental and physical health [3].

Research on EE dates back to the mid-20th century when Donald Hebb, a Canadian scientist, investigated the effects of enriched environments on rats’ behavior and cognition [4]. Hebb’s influential work, *The Organization of Behaviour: A Neuropsychological Theory* (1949), emphasized the role of experience in shaping behavior [5]. This laid the foundation for later studies examining the relationship between EE, brain plasticity, and behavior. In the 1960s, research shifted to exploring the effects of EE on dendritic branching in the brain, solidifying the concept that EE promotes brain development [6,7]. Subsequent contributions by Cummins et al. demonstrated that rats housed in enriched environments exhibited more significant brain growth than those in isolation [8]. During this period, EE was increasingly applied to the welfare of animals in captivity, highlighting its critical role in supporting both the physiological and psychological well-being of captive species [9]. By the 1990s, the American Zoo & Aquarium Association had incorporated EE into its guidelines, promoting its use to enhance wild animal welfare through principles of behavioral biology [10].

EE has continued to evolve, especially with advancements in science, technology, and neuroscience. For instance, Kim-McCormack et al. explored how interactive technologies, such as touch screens and motion-sensing devices, could improve animal well-being, particularly among great apes [11]. These technologies offer animals greater control over their environments, stimulating cognitive abilities and encouraging species-appropriate behaviors. Despite some reservations about non-natural stimuli, research has shown that digital stimulation can reduce stress and stereotypic behavior while promoting problem-solving and social interactions [11].

## 2. Domains of Environmental Enrichment

*Physical Enrichment*—Physical enrichment refers to the voluntary movement that involves skeletal muscles, leading to energy expenditure and promoting well-being [12]. In animals, this often involves providing exercise opportunities such as running wheels or toys, which have been shown to stimulate neurogenesis and increase brain plasticity, particularly in the hippocampus [13]. Physical activity also improves synaptic plasticity, which is essential for learning, memory, and overall mental health [14]. In practical applications, physical enrichment can reduce stress and enhance psychological well-being. For example, toys such as mazes or tunnels have been shown to stimulate exploration and reduce stress in rodents [15]. In human settings, physical enrichment can take the form of fitness programs or team sports, both of which have been linked to improved mental health outcomes [16,17]. In therapeutic contexts, cognitive–physical exercise interventions have been shown to enhance neuroplasticity in patients with traumatic brain injury or psychological disorders [18,19].

*Cognitive Enrichment*—Cognitive enrichment involves providing experiences that enhance cognitive functions, such as memory, problem-solving, and decision-making, by fostering neural plasticity. Cognitive enrichment is essential for developing areas of the brain involved in spatial learning (hippocampus) and complex cognitive processes (prefrontal cortex) [20]. In animals, cognitive enrichment often includes puzzle feeders or mazes that challenge problem-solving abilities and spatial memory [21]. Similarly, in humans, activities such as puzzles or educational classes stimulate cognitive flexibility and enhance mental functioning [22]. In both animal and human contexts, cognitive enrichment helps reduce boredom, fosters engagement in natural behaviors, and improves the quality of life. Practices such as mindfulness meditation or yoga have also been shown to enhance cognitive functioning by improving attention control and emotional regulation [23].

*Sensory Enrichment*—Sensory enrichment engages the brain through various sensory modalities, including sight, sound, smell, taste, and touch which promotes natural behaviors and improves mental well-being. For example, in animals, visual enrichment may involve using UV-reflective objects, while auditory enrichment could include species-specific sounds to encourage innate behaviors [24]. In humans, sensory activities such as music lessons or olfactory training can enhance cognitive functions, speech processing, and brain function [25]. In therapeutic settings with elderly individuals, sensory-based interventions, such as olfactory training and music therapy, are used to support cognitive development and rehabilitation which has been shown to improve emotional and cognitive well-being by sustaining cognitive function over time [26].

*Social Enrichment*—Social enrichment involves enhancing an individual’s environment through positive social interactions, which are critical for mental and cognitive growth. In animals, social enrichment often includes creating environments that encourage social connections and natural behaviors, such as larger cages or opportunities for social interaction [27]. In humans, social activities like group sports or community involvement have improved mental health and overall well-being. For example, support groups or social clubs were shown to provide emotional support and reduce feelings of isolation, aiding in stress management and improving mental health among high school students and the general community [28,29].

## 3. From Rodent Cages to Human Environments: The Environmental Enrichment Discrepancies

From the perspective of stroke rehabilitation, McDonald et al. identified several challenges in translating EE from animal preclinical studies to human interventions which in our view, encompass the general idea of EE translation [30].

### 3.1. The Disparity between the Environments of Laboratory Rodents and Humans

A significant barrier in applying EE from animal studies to human rehabilitation lies in the stark difference between environments. Laboratory rodents typically live in small cages with minimal stimulation, while humans generally inhabit enriched settings that naturally offer cognitive, physical, and social stimulation [31]. Some scholars suggest that EE is not an “enhancement” but rather a reversal of the impoverished conditions found in standard animal housing. Queen et al. argue that EE models human aging more accurately than standard housing, as it mimics the complex stimuli humans regularly encounter [32]. On the contrary, this perspective positions EE as an essential model for understanding neuroplasticity and brain recovery, especially when considering human variability in environmental exposure.

### 3.2. Challenges in Standardizing Environmental Enrichment Across Preclinical and Clinical Settings

In discussing the challenges of translating EE interventions from preclinical to clinical settings, several critical issues arise. One of the primary barriers is the difficulty in standardizing EE conditions across clinical sites. In preclinical settings, such as animal studies, creating and maintaining a standardized EE is relatively straightforward. Researchers can control and vary environmental factors like the novelty of objects and tasks and ensure that animals have unrestricted access to all areas within their cages. This control facilitates consistent EE experience across subjects, allowing for clearer interpretations of outcomes [30]. In contrast, translating these approaches to humans presents significant challenges.

For example, clinical environments for stroke rehabilitation are inherently diverse; variations in facilities, patient impairments, length of stay, and cost constraints all contribute to inconsistencies in EE implementation. For instance, stroke rehabilitation units can differ greatly in their physical setup and resources, which complicates efforts to standardize EE conditions. Patients’ access to various therapeutic activities may be limited by their physical impairments or the specific setup of their rehabilitation unit, and financial constraints often prevent frequent or extensive modifications to the environment [30].

The EE in animal studies often involves specific toys, shelters, and bedding that may not have direct counterparts in human rehabilitation. Each animal’s unique experience of EE—shaped by the specific combination of stimuli they encounter—further complicates efforts to replicate or adapt these conditions for humans. Similarly, while efforts have been made to replicate the physical, social, and cognitive aspects of enrichment from preclinical studies, such as exercise wheels for rodents, similar opportunities for intensive exercise are generally absent in clinical environments [30].

This discrepancy underscores the challenge of ensuring that the rich and varied stimuli provided in animal studies are appropriately mirrored in human settings, and it highlights the need for innovative solutions to bridge the gap between preclinical success and clinical application [30,32].

### 3.3. Unresolved Biological Mechanisms and Translational Challenges of Environmental Enrichment

While the benefits of EE have been observed in preclinical studies, the detailed biological mechanisms responsible for these effects are not yet fully understood [30]. Current research largely relies on correlational data, with a limited ability to pinpoint precise neurobiological pathways in human subjects. The tools available to study human brain mechanisms are more limited compared to those used in rodent research. For instance, while functional magnetic resonance imaging (fMRI) can provide valuable insights into brain activity by measuring blood flow changes, it is an indirect measure of neuronal activity and can be challenging to interpret [31].

Early-phase studies are crucial for deepening our understanding on how an enriched environment affects neurobiology and individual differences. While human research cannot directly probe biological mechanisms as preclinical studies do, insights from animal models can help to identify key biomarkers for clinical investigation. Despite the known conservation of certain EE mechanisms, such as brain-derived neurotrophic factor (BDNF), leptin, adiponectin, and the hypothalamic–pituitary–adrenal (HPA) axis stress response, between mice and humans, there may be additional mechanisms specific to human-centric stimuli [32]. If these mechanisms are conserved, EE models in rodents may offer promising translational benefits for humans. Conversely, differences between species could reveal new targets for future research and enhance the development of effective human interventions.

Furthermore, a limitation of current preclinical studies is that their focus was mostly on young male rodents, with little attention given to sex-specific differences or the effects across different life stages [30,33]. Future research should address these gaps by including both sexes and considering critical periods of the lifespan, which will be crucial for translating EE interventions into effective non-pharmaceutical treatments.

### 3.4. Limited Understanding of Which Specific Domains of Enrichment—Such as Physical, Social, Sensory, or Cognitive Stimulation—Are Crucial for Enhancing Brain Plasticity

In laboratory settings, animal enrichment typically includes access to exercise, additional social interactions, and environmental novelty. These elements are designed to boost mental stimulation and improve brain function [32]. However, the complex nature of human intelligence and social structures means that similar enrichment strategies may need to be more sophisticated or fundamental to achieve meaningful psychological and health benefits [30].

Furthermore, the significant role of stimulation and inspiration in promoting human well-being is exemplified by various initiatives, such as free or discounted access to public libraries, community centers, parks, and recreational facilities [32]. These resources support cognitive stimulation, physical activity, and social interaction. Similarly, some cities provide free or reduced admission to arts and cultural events, including concerts, theater performances, and art exhibitions, highlighting the importance of cultural engagement. Additionally, free or low-cost educational workshops and wellness programs contribute to intellectual and emotional enrichment. Unlike animal models, humans engage in complex careers, hobbies, and spiritual or religious practices, and possess a refined sense of auditory and visual aesthetics—elements that are difficult to replicate in animal studies. The perception of beauty and the impact of various stimuli can vary significantly between individuals and evolve over time, complicating the task of distinguishing between instinctual responses and personal preferences [32].

### 3.5. The Optimal “Dose” of Enrichment Remains Uncertain, as Most Laboratory Studies Use Continuous Periods of Enrichment, Which Is Seen as Impractical for Human Application

A review by Simpsons and Kelly (2011) highlights significant variation in the duration of EE regimens across animal studies where most of these studies employ a continuous EE paradigm, utilizing extended periods of enrichment without any breaks [34]. A significant portion of studies (40%) employ EE protocols lasting between 4 to 8 weeks, making this the most frequently used time frame. Additionally, 28% of the studies adopt a shorter 1–4 weeks duration, while 14% use protocols lasting 9–13 weeks. A smaller percentage of studies extend the EE duration beyond three months, with 5% lasting between 4 to 6 months and only 9% applying EE for a full year. Protocols lasting more than one year are rare, accounting for just 2% of the sample, while protocols lasting less than one year but more than six months also account for 2%. Although the precise lengths of time frequently appear to be selected at random, the comparability of study results is made more difficult by the lack of apparent standardization, raising concerns regarding the appropriate duration of EE exposure.

The optimal amount of enrichment necessary for effective brain plasticity remains unclear, as most laboratory studies use continuous periods of enrichment, which clinicians often consider impractical for real-world settings [35,36,37]. A clearer understanding of the ideal “dose” of enrichment is needed to translate these findings into clinical practice.

## 4. Overview of Short-Term or Restricted Daily Environmental Enrichment from Rodent Studies

To address the practical limitations associated with continuous EE, we reviewed the existing literature on daily short-term or restricted environmental enrichment (REE), particularly in rodent models. We conducted an online search using keywords such as “short-term enrichment”, “restricted enrichment”, and “daily enrichment” across databases including EBSCO, Semantic Scholar, Google Scholar, and PubMed. Inclusion criteria required original articles published in English that utilized rodent models in laboratory settings, while exclusion criteria removed review articles and studies involving farm or zoo animals. This search initially yielded 39 articles; after removing 6 duplicates and 12 articles that were either reviews or focused on continuous environmental enrichment, 21 original articles remained, all of which applied daily restricted or short-term EE paradigms in rodent experiments. Table 1 presents a range of studies investigating daily REE, focusing on factors like disease model, rodent species, research endpoint, duration of REE, and the enrichment setting.

### 4.1. Disease Models and Research Endpoints of Restricted Environmental Enrichment in Rodent Studies

**Figure 1:**
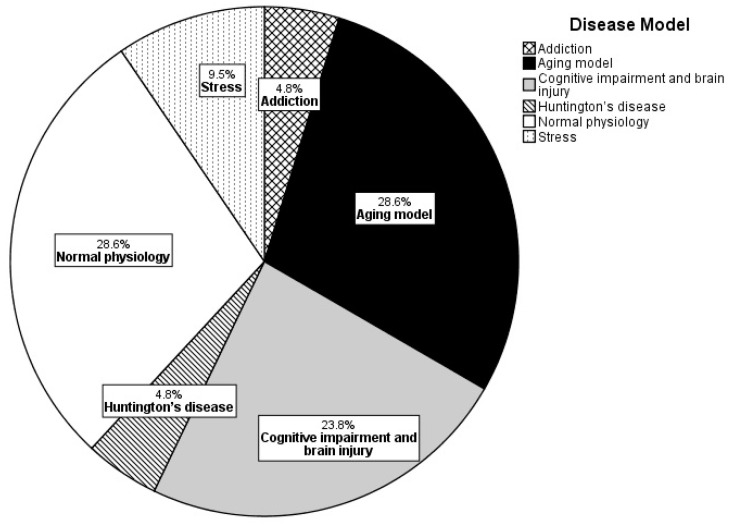
Pie chart depicts commonly employed disease models in REE rodent studies.

Aging and normal physiology models are the most frequently used in these studies, each constituting 28.6% of the research focus (Figure 1). Closely following these, models of cognitive impairment and brain injury represent 23.8%, highlighting a significant interest in the effects of REE on cognitive health. Stress models make up 9.5% of the studies, while addiction and Huntington’s disease are the least represented, each comprising only 4.8% of the research. Across these studies, the primary research endpoints focus on behavioral outcomes, with a particular emphasis on learning, memory, and stress-related behaviors. This focus reflects an interest in understanding cognitive and emotional responses stimulated by REE. In addition to behavioral assessments, research often investigates neurobiological processes, such as synaptic transmission, neuroplasticity, histology, and neurogenesis. A smaller subset of studies explores neurobiological aging, circadian rhythm, and neuroinflammation markers, which are less commonly addressed but remain relevant to understanding broader physiological impacts.

The high representation of aging and normal physiology models in REE studies suggests a strong interest among researchers in examining how daily short-term environmental stimulation impacts aging and overall health. This focus aligns with previous findings showing that environments deficient in stimuli can accelerate age-related cognitive decline and adversely affect physiological functions [56]. Moreover, interest in using REE in these models may also stem from the potential of adult neurogenesis to reveal the underlying neurobiology of this cognitive decline. Exploring neurogenesis within REE paradigms helps elucidate how aging affects the brain’s adaptability and response to environmental stimuli, as neurogenesis declines significantly with age.

The focus on cognitive impairment and brain injury rodent models in studies applying REE likely reflects an interest in exploring innovative rehabilitation strategies in order to optimize rehabilitation protocols, offering a practical and targeted approach to support recovery and improve outcomes. Interestingly, stress and addiction models are underrepresented in REE research, despite their relevance for examining the effects of REE, given the strong link between environmental factors and vulnerability to stress or addiction [57]. This limited focus may hinder a deeper understanding of how REE could improve these conditions, especially in clinical settings where environmental impoverishment can contribute to mental health disorders.

### 4.2. Settings and Duration of Restricted Environmental Enrichment in Rodent Studies

Most studies on REE do not provide detailed classifications of enrichment items according to specific enrichment domains, with notable exceptions being Rojas-Carvajal et al. and Lambert et al. [35,47]. The classification of enrichment items is crucial, as it allows for a clearer understanding of how different types of enrichment can influence various behavioral and cognitive outcomes. For instance, the domains of enrichment—such as social, physical, and cognitive—may have distinct effects on neuroplasticity and behavioral flexibility [48]. Additionally, only Konkle et al. and Gui et al. explicitly mention that they conducted their REE during the dark phase of the light/dark cycle [53,55]. This timing is crucial, as rodents are nocturnal animals, and the phase during which enrichment interventions are applied can significantly influence behavioral outcomes. Research has shown that nocturnal activity patterns affect cognitive performance and stress responses in rodents, highlighting the importance of considering circadian rhythms when designing behavioral interventions [58]. Therefore, the phase of enrichment delivery should be carefully reported and standardized in future studies to ensure the validity and reproducibility of findings related to cognitive and behavioral enhancements.

**Figure 2:**
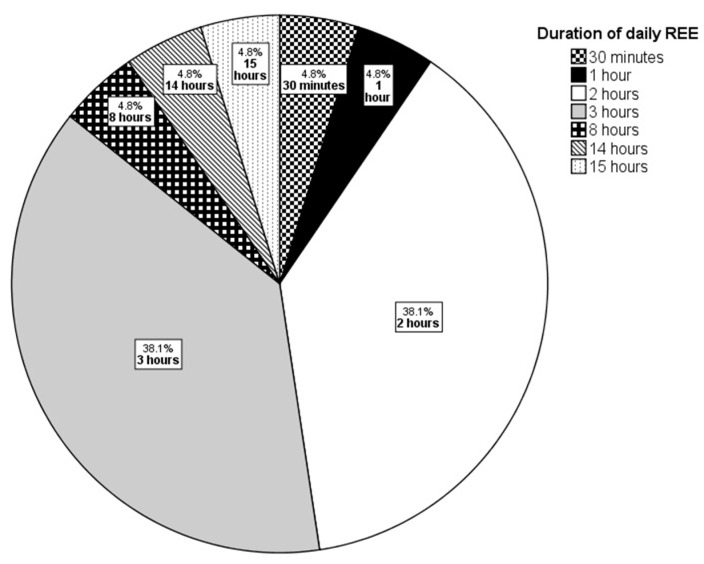
Pie chart depicts commonly employed daily REE duration in rodent studies.

The duration of REE varies widely across studies, from brief sessions of 30 min to extended exposures of up to 15 h per day. Notably, the rationale behind selecting specific durations is rarely detailed, which raises questions about the consistency and comparability of findings across studies. Among the 21 studies reviewed, the most common durations for REE are 2 h (38.1%) and 3 h (38.1%) daily (Figure 2). These timeframes provide valuable insights into REE’s effects on cognitive performance, neuroplasticity, and stress resilience in rodent models across various research domains.

#### 4.2.1. Cognitive and Neural Benefits of 2-h Daily REE

Research on 2 h daily REE demonstrates that even brief enrichment sessions can significantly impact brain structure and function. A foundational study by Rosenzweig et al. [36] found that 2 h of daily REE over 55 days led to a notable increase in cerebral cortex weight in male rats, alongside elevated levels of key enzymes, including acetylcholinesterase (AChE) and cholinesterase (ChE). Additionally, an increase in the cortical-to-subcortical brain ratio underscored how limited enrichment could swiftly impact cortical development, laying the groundwork for understanding the cognitive benefits associated with shorter REE sessions.

Building on Rosenzweig’s findings, subsequent studies documented behavioral and cognitive advantages of 2 h REE. Widman et al. [37,40] observed that 2 h sessions enhanced exploratory behavior, with rats in enriched environments showing greater interaction with novel objects than those in impoverished settings. This suggests that restricted enrichment can cultivate curiosity and adaptability, yielding cognitive benefits similar to those of longer protocols. These 2 h sessions also improved performance on learning tasks without increasing sensitivity to uncontrollable stress, indicating that limited REE bolsters basic cognitive functions without causing overstimulation.

#### 4.2.2. Neurodevelopmental Effects and Recovery with 2-h REE

In the context of neurodevelopment, Ji et al. [43,44] showed that 2 h REE alleviated cognitive and synaptic impairments due to neonatal sevoflurane exposure. Across 34 to 82 days, daily enrichment significantly enhanced hippocampal function and normalized interneuron profiles, emphasizing REE’s role in supporting neuroplasticity and recovery. Similarly, Rojas-Carvajal et al. [35] found that restricted and unpredictable enrichment positively influenced neurobehavioral outcomes, demonstrating that even brief enrichment can enhance neural resilience.

However, REE’s impact on behavior can vary with context. For instance, Fukushiro et al. [41] reported that intermittent 2 h REE sessions during amphetamine treatment exacerbated addiction-related behaviors, emphasizing the need to carefully consider the behavioral context when implementing REE, as it may support cognitive functions while inadvertently promoting maladaptive behaviors.

#### 4.2.3. Enhanced Memory and Synaptic Function with 3-h Daily REE

Research on 3 h of daily REE shows significant benefits for memory, synaptic function, and cognitive recovery in diverse contexts, including age-related decline and mental health. Rampon et al. [45] demonstrated that 3 h of daily enrichment over two months could reverse memory deficits in NMDA receptor knockout mice, with an increase in synapse density in the CA1 region, suggesting that REE can enhance memory even with molecular impairments. These findings expand on foundational studies by Frick et al. [46] and Lambert et al. [47], who explored 3 h REE’s effects on memory and synaptic changes in both young and aged mice.

Frick’s work highlighted that 23 days of REE could mitigate age-related spatial memory deficits and elevate synaptic protein levels, indicating that moderate enrichment durations promote cognitive resilience in older animals. Lambert’s research added depth by examining different REE components, such as exercise and cognitive stimulation, which improved working memory and promoted neuroplasticity. Together, these studies reinforce that 3 h of daily enrichment effectively enhances memory across age groups.

While Bennet et al. [48] emphasized continuous enrichment for substantial synaptic recovery, Sampedro-Piquero et al. [49] found that 3 h of daily enrichment for two months improved spatial memory in both young and aged rats, with younger animals showing more pronounced benefits. This suggests that, although shorter REE sessions enhance cognitive function in all age groups, younger populations may experience greater gains, while older animals might benefit more from continuous and complex enrichment for robust recovery.

#### 4.2.4. Additional Findings: Age-Related Synaptic Decline, Mental Health, and TBI Recovery

Studies on age-related synaptic decline support the benefits of 2–3 h of REE. Speisman et al. [42] and Stein et al. [51] demonstrated that daily enrichment effectively reverses cognitive and synaptic declines in aged rats. Speisman et al. found that REE promotes new neuron survival despite age-related reductions in neurogenesis, facilitating flexible spatial information use. Similarly, Stein et al. showed that 3 h daily enrichment over three weeks restored hippocampal synaptic function in aged rats, highlighting that lifelong enrichment may not be necessary to counteract synaptic aging.

In mental health, Ramírez-Rodríguez et al. [52] found that 3 h of daily REE combined with fluoxetine treatment reversed depressive-like behaviors and hippocampal neurogenesis deficits in chronically stressed mice, demonstrating REE’s potential as a supplement to pharmacological treatments.

Lastly, Radabaugh et al. [50] investigated REE’s effects on traumatic brain injury (TBI) recovery, finding that two 3 h REE sessions per day yielded comparable improvements in motor function and spatial learning to a single 6 h session. This suggests that session structure—whether divided or continuous—does not significantly affect recovery, underscoring the efficiency and adaptability of 3 h REE regimens.

#### 4.2.5. Time Efficiency in REE Protocols for Practical Research Settings

In summary, both 2 h and 3 h daily REE yield significant cognitive and neural benefits without requiring longer sessions, making them applicable in practical research settings where time constraints or limited access to EE environments may preclude extended sessions. Notably, 2 h REE appears particularly suitable for studies on neurodevelopmental disorders and recovery from stress or injury, whereas 3 h REE is more often used in age-related studies focusing on memory and neurogenesis. These findings collectively underscore that substantial neurobehavioral improvements can be achieved with restricted enrichment durations, offering flexibility for diverse research contexts.

## 5. Real-World Applications of Restricted Daily Environmental Enrichment in Human Settings

Interestingly, daily REE programs have already been widely applied in human settings, often without us realizing it. These programs are designed to enhance cognitive and neurobehavioral functions and have been seamlessly integrated into our daily lives through various activities across different age groups.

For children and teenagers, REE is frequently seen in after-school enrichment classes, extracurricular activities, and summer camps. These environments often offer holistic experiences, engaging multiple aspects of learning, physical activity, and creativity. Structured enrichment programs during these formative years have been shown to improve cognitive abilities and promote overall well-being [59,60].

Meanwhile, adults tend to engage in more selective enrichment activities. Weekly enrichment programs, rather than daily, are often more feasible due to time constraints and other responsibilities. Studies suggest that engaging in cognitively stimulating activities, such as learning new skills or exercising, can improve mental health and delay cognitive decline in adulthood [61]. For the elderly, particularly in nursing homes or structured environments like maternity confinement centers, these enrichment activities can be more closely monitored, providing consistent cognitive and emotional benefits.

However, applying restricted daily REE to populations with specific needs, such as stroke patients, individuals with psychiatric conditions, or those recovering from neurological trauma, presents unique challenges. These groups often have complex, heterogeneous profiles, including varying degrees of cognitive and physical impairments, different medical histories, and distinct rehabilitation needs. This diversity makes it difficult to implement standardized REE programs that would be equally effective for all.

Effective enrichment in stroke rehabilitation hinges on introducing novelty and complexity, which can be challenging in inpatient settings where physical, social, and cognitive activity options are often limited. Stroke patients may experience a range of cognitive and motor deficits—such as attention, memory, language, and motor function impairments—based on the severity and location of the stroke. This diversity in impairments necessitates individualized rehabilitation approaches, making a one-size-fits-all model impractical [30]. To improve enrichment and cater to specific needs, it is essential to incorporate activities beyond traditional therapy. Integrating self-directed exercises and leveraging technology—such as gaming, robotics, and virtual reality—can enhance accessibility and effectiveness, offering tailored solutions that address the varied needs of stroke survivors [62].

In the case of individuals with psychiatric conditions, the challenges are equally complex. Patients with conditions like schizophrenia, depression, or anxiety may need a different set of enrichment activities, aimed not only at enhancing cognitive functions but also at improving emotional regulation and social engagement. For example, in psychiatric care settings, art therapy and music therapy have been shown to stimulate cognitive engagement and improve mood. Still, their effects can vary depending on the individual’s condition [63]. Implementing a generalized program could under-stimulate or overwhelm certain individuals, thereby reducing its effectiveness [64].

Moreover, the settings in which these populations receive care, such as hospitals, psychiatric facilities, or rehabilitation centers, add another layer of complexity. Stroke patients in acute care may not have the mobility or stamina for intensive daily enrichment, while psychiatric patients in controlled environments may have limited access to enriching stimuli, such as social interaction or physical activity. This limits the scope of standardized REE programs and emphasizes the need for flexible, individualized enrichment protocols.

Despite these challenges, tailored REE programs show significant potential for improving cognitive function and recovery. Research indicates that cognitive and physical enrichment in stroke patients enhances neuroplasticity, aiding in motor skill recovery and improving cognitive outcomes [65]. Similarly, in psychiatric populations, individualized interventions like structured social activities or creative therapies can reduce symptoms and improve overall functioning [66]. Developing personalized enrichment strategies—adjusting the intensity, type, and frequency of activities to suit individual needs—can therefore better address the unique impairments found in these populations.

## 6. The Future of REE: Making Environmental Enrichment Work at Home

One of the next critical gaps to address in daily REE is the development of programs that can be applied effectively in home settings. While most REE interventions have traditionally been implemented in specialized centers—such as rehabilitation facilities, nursing homes, or clinics—there is growing demand for enrichment activities that can be seamlessly integrated into everyday life without requiring patients to visit a facility. This shift toward home-based REE could greatly improve accessibility, making it possible for individuals to engage in cognitive and physical enrichment in the comfort of their homes.

Designing REE for home use presents both opportunities and challenges. On one hand, enabling patients to participate in enrichment activities at home removes barriers related to transportation, scheduling, and the need for supervision. This would be particularly beneficial for elderly individuals, those with mobility limitations, or individuals who live far from rehabilitation centers. Home-based REE could empower individuals and caregivers to take an active role in maintaining cognitive and physical health.

However, creating effective home-based REE programs requires careful consideration of the individualized nature of these interventions. While rehabilitation centers offer controlled environments with trained professionals to guide activities, home settings vary widely in terms of available resources, space, and support. Standardization across different cases is virtually impossible, as each individual’s condition, home environment, and access to technology and materials differ greatly. For instance, a stroke patient recovering at home may require specialized equipment for motor skill recovery, while a psychiatric patient might benefit more from social or creative activities that do not require tools but do need guidance.

The solution may lie in creating flexible, adaptable enrichment programs that provide options for varying levels of engagement and complexity. For example, digital platforms or mobile apps could offer cognitive exercises, memory games, or guided physical activities tailored to the user’s needs and capabilities. Virtual reality (VR) environments or online support communities could further enhance the experience by simulating more immersive environments akin to what might be available in specialized centers [67]. Caregivers or family members could be trained to facilitate these activities, helping to bridge the gap between home-based and professional-guided enrichment.

While standardization may be impossible, the key to success in home-based REE will be the development of customizable programs that allow for individual adjustment. This could include providing a menu of activities with different levels of intensity, difficulty, and focus—ranging from physical exercises to social engagement tasks and cognitive stimulation. By offering flexible, personalized options, home-based REE can be tailored to the needs and abilities of each participant without compromising its therapeutic potential.

The benefits of a well-designed home-based REE program are significant. It could reduce healthcare costs, alleviate the burden on rehabilitation centers, and most importantly, provide continuous, consistent cognitive and physical stimulation for individuals who might otherwise struggle to access these services. With advances in technology and a focus on individualization, home-based REE has the potential to become a vital tool in maintaining cognitive health and enhancing recovery outcomes.

## 7. Conclusions

Despite the challenges of translating REE from controlled animal studies to human environments, the research indicates that REE offers a flexible, time-efficient alternative to continuous enrichment, making it more feasible for clinical and everyday settings. Future research should continue to explore the optimal duration and structure of REE, particularly in human rehabilitation contexts. Additionally, the development of customizable, home-based REE programs could broaden the accessibility of these interventions, providing cognitive and physical enrichment to diverse populations. The consistent benefits observed from 2 and 3 h REE protocols in rodent studies point to a scalable model that has the potential to enhance well-being, even with limited daily exposure.

## Figures and Tables

**Table 1:** Restricted environmental enrichment in rodent studies.

Reference	Disease Model	Rodent Species	Research Endpoint	Duration of REE	Enrichment Setting
[38]	Aging model	Long-Evans male rats	Stress behavior, Neurobiology of inflammation	30 min (3 days/week, total of 5 weeks)	One meter in diameter circular open field. Three sections: a climbing area, a concealing area, and a play area. No mention of alternate changes in the apparatus settings.
[39]	Prenatal hypoxia-ischemia	Male Wistar rat	Reference memory, Working memory, Histology	1 h daily (6 days/week, total of 9 weeks)	A 40 × 60 × 90 cm cage. Three doors, ramps, a running wheel, and a variety of objects with various textures and shapes. Objects were changed once a week.
[36]	Normal model	Male Berkeley S3 strain rat	Neuroanatomy, Neurobiology of synaptic transmission	2 h, 2.5 h, 4.5 h (total of 55 days)	A large cage. Metal boxes, ladders, and running wheels, Hebb–Williams maze device. Objects and maze patterns were changed daily.
[40]	Normal model	Male Sprague Dawley rats	Exploratory Behavior	2 h (total of 1 month)	A 75 × 75 × 40 cm cage. At least 10 enrichment objects at a time. A total of 3 objects were switched from a pool of 20 enrichment objects daily.
[37]	Uncontrollable stress model	Male Sprague Dawley rats	Learning behavior	2 h (total of 40 days)	Same setting as Widman et al. [40]
[41]	Addiction model	Adult male Swiss mice	Addiction behavior	2 h (total of 13 days)	Four enrichment items were put and arranged randomly in the animals’ home cages. A little plastic house, a plastic tunnel, a chewable wooden object, and a new toy (PVC pipes, rubber balls, rubber rings, porcelain objects, etc.). A new toy of a different color and shape was added daily, while the other items were rearranged.
[42]	Aging model	Young and aged male Fischer 344 rats	Spatial learning and memory, Neurogenesis	2–3 h (total of 10 weeks)	Three-dimensional toys, a huge wooden box, an empty water maze tank, or a large wire cage. The arrangement of the items was changed daily.
[43]	Sevoflurane-induced cognitive impairment model	Male C57BL/6 mice	Learning behavior, Neuroplasticity	2 h (total of 34 days)	A 70 × 45 × 40 cm cage. The description of enrichment items was not mentioned. The arrangement of the items was changed two to three times per week.
[44]	Sevoflurane-induced cognitive impairment model	C57BL/6 male pups	Learning behavior, Memory behavior, Neurobiology of synaptic transmission	2 h (total of 82 days)	A 60 × 50 × 40 cm cage. The description of enrichment items was not elaborated but was referred to previous studies. The arrangement of the items was changed two to three times per week.
[35]	Normal model	Male Wistar rats	Exploratory Behavior, Neuroplasticity	2 to 48 h unpredictably (total of 1 month)	Wooden, natural enrichment items of four main categories (dens and hideouts, sensorimotor and physical stimuli, nesting and chewing materials, and highly palatable foods). The arrangement of the items was changed twice per week.
[45]	Genetically modified model	CA1 specific NMDA receptor 1 subunit knockout (CA1-KO) mice	Learning behavior, Neuroplasticity	3 h (total of 2 months)	A 1.5 × 0.8 × 0.8 m cage. Several toys, running wheels, and small houses were placed. The arrangement of the items was changed daily.
[46]	Aging model	Young and aged female C57BL/6 mice	Learning behavior, Neurobiology of synaptic transmission	3 h (total of 23 days)	A 56.5 × 41.5 × 22 cm cage. A running wheel, rodent toys (plastic boat, balls, seesaw, Critter Trail Puzzle Playgrounds), PVC pipe fittings in various shapes, and a toy rope suspended in different configurations. The arrangement of the items was changed daily.
[47]	Normal model	Young female C57BL/6 mice	Spatial learning and memory, Neurobiology of synaptic transmission	3 h (total of 6 weeks)	**Cognitive**: The 56.5 × 41.5 × 22 cm cage contained various objects, (toys, PVC pipes, hollow metal cylinders, Legos, and a toy rope). Running wheels were excluded, and four to five objects were rearranged daily. **Exercise**: Each cage had three running wheels (11.5 cm diameter), repositioned daily. **Acrobatic**: Ten bridges connected six wooden platforms (10 × 10 cm, 34.5 cm high) in two rows. The bridges were made from chains, rubber bands, metal rods, wires, and ropes, with changes made weekly.
[48]	Aging model	Young and aged male C57BL/6 mice	Spatial learning and memory, Neurobiology of synaptic transmission	3 h (total of 10 weeks)	A 56.5 × 41.5 × 22 cm cage. A running wheel, a plastic rodent dwelling, a plastic tube setup for vertical climbing, and two or three additional toys. The arrangement of the items was changed daily.
[49]	Aging model	Young and aged male Wistar rats	Spatial learning and memory, Neurobiology of aging	3 h (total of 2 months)	A 100 × 95 × 54 cm cage. Toys, running wheels, ropes, plastic tubes of varying diameters, platforms, wooden houses, objects with distinctive scents and sounds, and nesting materials. The configuration of the cage was changed once a week.
[50]	Traumatic brain injury model	Adult male Sprague- Dawley rats	Neurological function, Histology	3 h (twice) or 6 h (total of 21 days)	A 91 × 76 × 50 cm three-story cage. Toys (balls, blocks, and tubes), nesting materials. No mention of alternate changes in the apparatus setting.
[51]	Aging model	Male Sprague–Dawley rats	Neuroplasticity	3 h (total of 3 weeks)	A 1500 × 3000 × 3000 mm two-story cage. Toys and various objects. The arrangement of the items was changed daily.
[52]	Chronic mild stress model	Balb/C female mice	Stress behavior, Neurogenesis	3 h (total of 4 weeks)	A 34 × 44 × 20 cm cage. Colorful tunnels in different shapes, two running wheels, wooden pieces, nesting materials, and plastic houses with stairs. The complexity of enrichment items was modified every three days. EE was performed during the dark phase of the light/dark cycle.
[53]	Normal model	Male Long Evans and Sprague– Dawley rats	Stress behavior, Neurobiology of stress	8 h (5 days/week, total of 6 weeks)	A 0.73 × 0.44 wide × 0.75 m three-story cage. Grid sides for climbing, a metal running wheel, climbing rope, cloth hammock, large plastic tubes, and various small rubber and plastic toys. No mention of alternate changes in the apparatus setting.
[54]	Huntington’s disease model	R6/2 mice	Cognitive functions, Neuroanatomy	14 h (total of 8, 12, 16, 22 weeks)	A 60 × 30 × 45 cm cage. Ropes, ladders, running wheels, and toys. The arrangement and combination items were changed daily.
[55]	Postoperative cognitive dysfunction (POCD) model	Male C57BL/6	Cognitive functions, Neurobiology of circadian rhythm	15 h (total of 2 weeks)	A 43.18 × 22.86 ×19.05 cm cage. A running wheel, sheds, tunnels, and various toys. The arrangement of the items was changed twice per week. Exposure to EE occurred between 6 PM to 9 AM.

## Data Availability

This research does not contain new data.
